# Treatment of opioid withdrawal in neonates with morphine, phenobarbital, or chlorpromazine: a randomized double-blind trial

**DOI:** 10.1007/s00431-019-03486-6

**Published:** 2019-11-06

**Authors:** Urs Zimmermann, Christoph Rudin, Angelo Duò, Leonhard Held, Hans Ulrich Bucher, On behalf of the Swiss neonatal abstinence syndrome study group

**Affiliations:** 1 Department of Neonatology, Spital Bülach, CH–8180 Bülach, Switzerland; 2 General Pediatrics, University Children’s Hospital Basel, CH-4031 Basel, Switzerland; 3 Institute for Epidemiology, Biostatistics and Prevention, University of Zurich, CH-8001 Zurich, Switzerland; 4 Department of Neonatology, University Hospital Zurich, CH - 8091 Zurich, Switzerland

**Keywords:** Neonate, Withdrawal, Opioids, Pharmacological treatment, Morphine, Chlorpromazine, Phenobarbital

## Abstract

Three suitable compounds (morphine, chlorpromazine, and phenobarbital) to treat neonatal abstinence syndrome were compared in a prospective multicenter, double-blind trial. Neonates exposed to opioids in utero were randomly allocated to one of three treatment groups. When a predefined threshold of a modified Finnegan score was reached, treatment started and increased stepwise until symptoms were controlled. If symptoms could not be controlled with the predefined maximal dose of a single drug, a second drug was added. Among 143 infants recruited, 120 needed pharmacological treatment. Median length of treatment for morphine was 22 days (95% CI 18 to 33), for chlorpromazine 25 days (95% CI 21 to 34), and for phenobarbital 32 days (95% CI 27 to 38) (*p* = ns). In the morphine group, only 3% of infants (1/33) needed a second drug; in the chlorpromazine group, this proportion was 56% (24/43), and in the phenobarbital group 30% (13/44).

*Conclusion*: None of the drugs tested for treating neonatal abstinence syndrome resulted in a significantly shorter treatment length than the others. As morphine alone was able to control symptoms in almost all infants, it may be preferred to the two other drugs but should still be tested against more potent opioids such as buprenorphine.

*Trial registration*: At ClinicalTrials.gov NCT02810782 (registered retrospectively).

**Table:** 

**What is Known:** • *Neonates exposed to opiates in utero and presenting with withdrawal symptoms should first be treated by non-pharmacological supportive measures.* • *In those who fail, drugs have to be given, but there is controversy which drug is best.*	
**What is New:** • *Among three candidates, morphine, chlorpromazine and phenobarbital, none resulted in significantly shorter treatment time.* • *As morphine alone was able to control symptoms in almost all infants, it may be preferred to the two other drugs.*

**What is Known:**

• *Neonates exposed to opiates in utero and presenting with withdrawal symptoms should first be treated by non-pharmacological supportive measures.*

• *In those who fail, drugs have to be given, but there is controversy which drug is best.*

**What is New:**

• *Among three candidates, morphine, chlorpromazine and phenobarbital, none resulted in significantly shorter treatment time.*

• *As morphine alone was able to control symptoms in almost all infants, it may be preferred to the two other drugs.*

## Introduction

The incidence of withdrawal symptoms in newborns exposed to opioids during pregnancy, termed neonatal abstinence syndrome, has increased in the last 20 years and has reached epidemic proportions in high-income countries (2–6/1000 live births) [2]. Neonatal abstinence syndrome not only poses an important burden of suffering on infants and families but also contributes to the occupancy of neonatal beds and generates considerable health care costs [5].

There is consensus that management of neonatal abstinence syndrome should primarily be focused on reducing symptoms of withdrawal such as hyperirritability, excessive crying, poor feeding, vomiting, and diarrhea [12]. In the first instance, non-pharmacological supportive measures are indicated, such as quiet environment and frequent feeds to ensure sufficient caloric intake [10]. However, in up to 60% of infants with neonatal abstinence syndrome, pharmacological therapy has to be given to control persistent neurological and gastrointestinal symptoms [13].

The present study was designed in the late 1990s as part of a national program addressing detoxification supported by the Swiss Health Agency. At that time, several substances were used to treat neonatal abstinence syndrome, but few randomized controlled studies were available, and there was no consensus about the best medication or the optimal dose [8]. Morphine and phenobarbital were most widely used in the USA [21]. Chlorpromazine was most commonly used in the UK for its effect on the central nervous system and the gastrointestinal system [17]. Phenobarbital was mainly used with loading and maintenance doses for its sedative and anticonvulsant properties [19].

The goal of the trial reported here was to compare three substances in a double-blind multicenter trial for the treatment of neonatal abstinence syndrome.

## Methods

This was a multicenter, double-blind, parallel-group study with three arms conducted in seven neonatal intensive care units in Switzerland.

The main objective was to document the effect of three drugs (oral morphine solution, chlorpromazine, phenobarbital) on the length of treatment based on the modified Finnegan score. Secondary objectives were to document the need for a second drug, the occurrence of seizures, and other adverse events. We included late preterm and term infants (34 gestational weeks or more) who had withdrawal symptoms and were born to mothers who took opioids, including methadone, during pregnancy. We excluded infants with diseases probably requiring a long hospitalization.

All infants with reported maternal opioid consumption and suspected neonatal abstinence syndrome were assessed with a modified Finnegan score every 8 h, the standard interval in Swiss Hospitals [23]. If an infant scored once above 14 or twice in a row above 9, and the parents had given written consent, the infant was randomized to group A (morphine), B (chlorpromazine), or C (phenobarbital).

The following possible confounders for the primary outcome were collected: hospital, gestational age at birth, birth weight, head circumference at birth, sex, pH in umbilical artery, mode of delivery, Apgar score at 5 min of life, postnatal intervention, socioeconomic status of the parents (scale 2–12, 2 = highest status [14]), and drugs detected in the meconium.

### Preparation and administration of study drug (Fig. 1)

Vials with the study drug were prepared in the pharmacy of Zurich University Hospital according to a computer-generated randomization list, labelled, shipped, and stored according to the Swiss Therapeutic Product Act of 15 December 2000. In order to mask group allocation, water, ethanol, glycerine, and caramel color (E150) were added to the active substance, and dosing regime was standardized. Since phenobarbital treatment needs a loading dose and morphine and chlorpromazine do not, each of the three substances was prepared in two distinctive vials. A red-labelled vial contained the solution to be given as a starting dose: 0.25 ml of the solution contained either 0.25 mg morphine or 0.5 mg chlorpromazine or 10 mg phenobarbital. White-labelled vials with the standard solution of morphine and chlorpromazine were identical to the starting solution, while the standard solution of phenobarbital contained 0.83 mg/0.25 ml. If pharmacological treatment was indicated, the infant first received a dose of 0.25 ml per kilogram body weight of the starting solution (red-labelled vial), followed by 0.25 ml per kilogram body weight of the white-labelled standard solution every 4 h. The drugs were given orally or by nasogastric tube. Physicians, nurses, and parents were blinded.

**Fig. 1:**
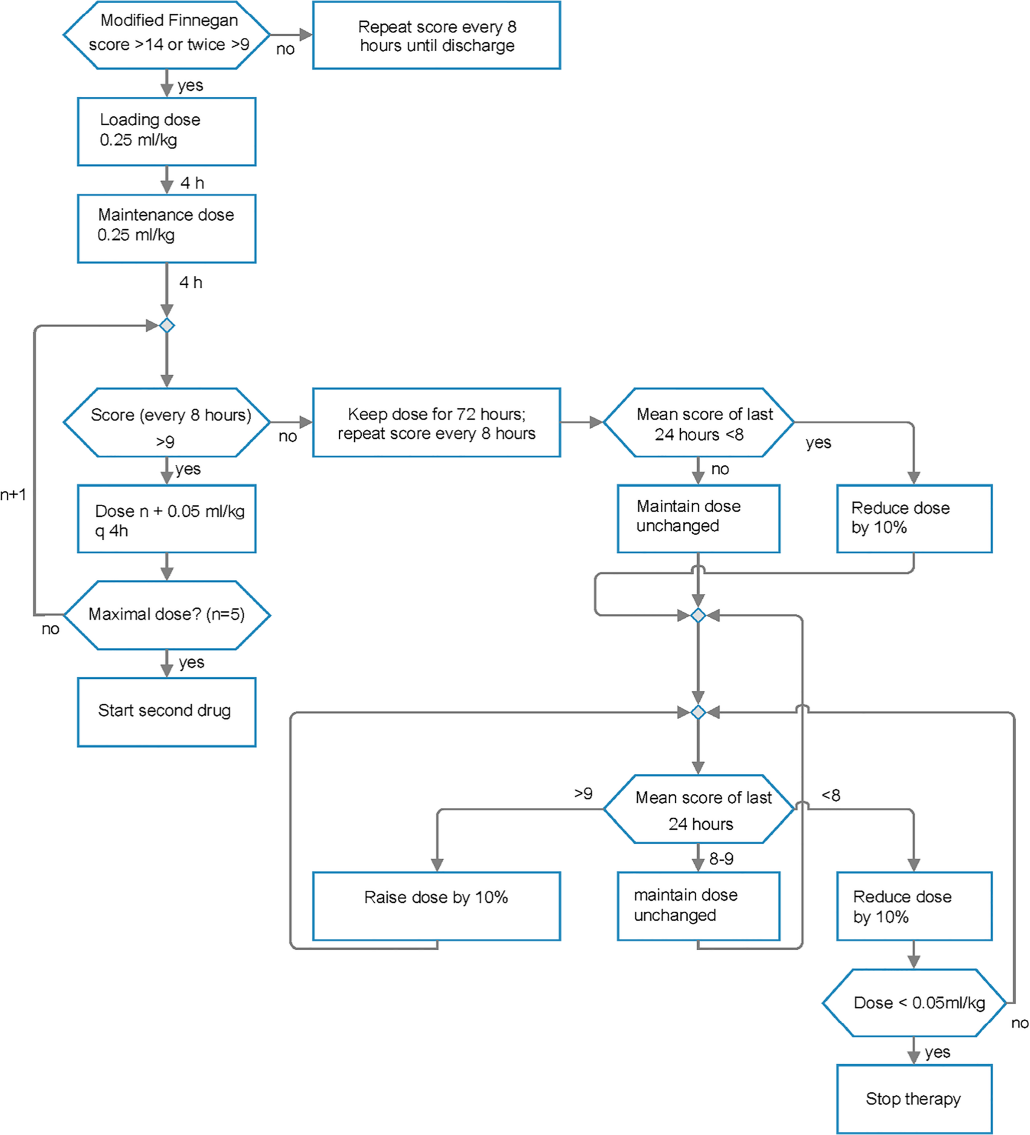
Therapy algorithm. Medications are started, increased, decreased, or stopped depending on a modified Finnegan score. If symptoms were not controlled by the predefined maximal dose of the first allocated drug, a second drug was added following the same algorithm as the first drug

### Increase of dose

After initiation of therapy (see above), a modified Finnegan score was recorded every 8 h [23]. Each time the score was above 9, the study drug was increased by 0.05 ml/kg until a maximum dose of 0.5 ml/kg was achieved or the withdrawal symptoms were controlled. The symptoms were judged to be controlled if the modified Finnegan score was below 9 on three consecutive measurements. If symptoms were not controlled by the defined maximum dose of the allocated drug, a second drug was added following the same algorithm as the first drug. The first drug was continued at the maximal level. The predefined maximum doses of the drugs led to daily dosages of 3 mg/kg for morphine, 3 mg/kg for chlorpromazine, and 10 mg/kg for phenobarbital. The second drug was predefined by allocation of the first drug and also blinded. If the first drug was morphine, the second drug was phenobarbital. If the first drug was phenobarbital or chlorpromazine, the second drug was morphine.

### Reduction of dose

If symptoms were controlled, the allocated drug or the combination of two drugs was administered at the same dose for the next 72 h. After this stabilization period, the drugs were reduced stepwise. If the mean modified Finnegan score for the last 24 h was below 8, the drug was reduced by 10%. If the mean score was 8 or 9, the drug dose was kept unchanged, and if the mean score was above 9, the drug was increased again to the previous level. If two drugs had to be given, the first drug was reduced first. If the infant needed no more drugs for 2 days, it could be discharged.

### Monitoring

The infants were routinely assessed clinically: modified Finnegan score every 8 h, clinical examination and weight daily. Two samples of meconium were analyzed for opioids, methadone, amphetamine, barbiturate, benzodiazepine, cocaine, and cannabis. Blood glucose and electrolytes were investigated only on clinical indication.

*Suffering* was quantified with the sum of modified Finnegan scores above 9 for the whole treatment period. *Intensity of care* was documented continuously based on how much time nurses spent with one infant, documented in intervals of 15 min during 24 h.

### Sample size calculation

The primary objective of this study was the length of pharmacological treatment. A retrospective analysis of 90 neonates born to opioid-addicted mothers in the University Hospital Zurich over a period of 10 years found that the mean length of treatment of infants with neonatal abstinence syndrome was 30 days with a SD of ± 12 days. We considered a difference of more than 1 week (8 days or more) to be clinically and economically important. To detect a difference of 8 days between two groups, we calculated a minimal sample size of 36 (alpha 0.05, power 80%) per group.

### Statistical methods

The treatment time and hospitalization time were analyzed using a Kaplan-Meier time-to-event analysis. The resulting curves were then compared using the Peto modification of the log-rank test. To adjust for covariates, a Cox proportional hazard regression and Weibull models were used, the latter allowing for the computation of event–time ratios [4]. The need for a second drug was analyzed using logistic regression and Fisher’s exact test.

The global *p* value for an effect of treatment between the model with and without the treatment term was assessed with a likelihood ratio test (LR). *Suffering*, defined as the sum of modified Finnegan scores > 9, was analyzed using a linear model with the scores as the dependent variables, which were log10 transformed to guarantee normality and a constant variance of the residuals. Similarly, a linear model was used to analyze the intensity of care.

The association of the drugs found in the meconium with treatment length was tested using univariable linear models and a multivariable linear model.

The study protocol was approved by the Ethics Review Board of the Canton Zurich and by the Swiss Agency for Therapeutic Products (StV Nr. 11/00). All parents gave written informed consent to participate in the study.

## Results

**Fig. 2:**
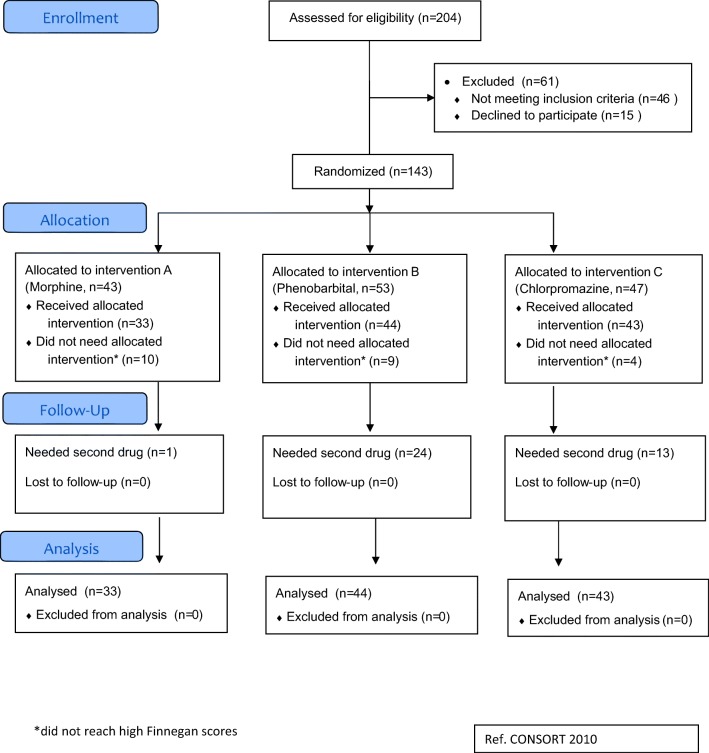
Flow diagram (reference CONSORT 2010)

A total of 204 infants born to a mother who reported taking opioids, including methadone, during pregnancy were assessed for eligibility in the seven participating hospitals (see flow diagram in Fig. 2). Of these, 61 infants were excluded for not meeting all inclusion criteria, of whom 15 were because parents refused consent.

Among the 43 infants allocated to intervention A (morphine), 10 did not reach the criteria for treatment during the whole observation period and therefore did not receive the allocated drug. In intervention B (chlorpromazine), 9 out of 53 infants and in intervention C (phenobarbital), 4 out of 47 infants did not need treatment and consequently were not included in the analysis.

The three study groups were well balanced, and there was no difference in sex, gestational age, birth weight, head circumference, mode of delivery, Apgar score at 5 min, pH in umbilical artery, need for postnatal intervention or socioeconomic status of the parents (Table 1).

**Table 1:** Group characteristics

	Morphine (*n* = 43)		Chlorpromazine (*n* = 53)		Phenobarbital (*n* = 47)		*p*	Test
Sex								
Female	19	44.2%	25	47.2%	23	48.9%	0.902	Chi-square
Male	24	55.8%	28	52.8%	24	51.1%		
Gestational age (weeks, median [IQR])	38.14	[37.0, 38.93]	38.36	[37.14, 38.0]	39.0	[38.0, 40.0]	0.073	ANOVA
37 (0/7)–42 (0/7)	34	79.1%	43	82.7%	44	93.6%		
34 (0/7)–36 (6/7)	9	20.9%	9	17.3%	3	6.4%		
Birth weight (g, median [IQR])	2700	[2452, 3070]	2765	[2443, 3100]	2800	[2540, 3070]	0.646	ANOVA
Small for GA (< 10th percentile)	18	41.9%	20	37.7%	19	40.4%		
Head circumference (cm, median [IQR])	33.0	[32.0, 34.5]	33.0	[32.0, 34.0]	33.0	[32.0, 33.5]	0.765	ANOVA
Microcephaly (< 10th percentile)	11	25.6%	19	35.8%	13	27.7%		
Mode of delivery							0.32	Chi-square
Spontaneous	16	37.2%	28	53.8%	27	57.4%		
Cesarean section	21	48.8%	17	32.7%	16	34.0%		
Vacuum or forceps	6	14.0%	7	13.5%	4	8.5%		
Apgar 5 min (median [IQR])	9	[9, 9]	9	[9, 9]	9	[9, 9]	0.829	Kruskal
< 6	0	0.0%	1	2.1%	1	2.4%		
pH in umbilical artery (median, [IQR])	7.27	[7.22, 7.33]	7.28	[7.23, 7.3]	7.25	[7.21, 7.29]	0.353	ANOVA
NA-pH < 7.15	1	2.4%	3	6.5%	2	4.8%		
Postnatal intervention (bag and mask or supplemental oxygen)	3	7.0%	2	3.8%	2	4.3%	0.746	Chi-square
Socioeconomic status of parents (median [IQR])	8	[7, 9]	7	[6, 8]	7	[6, 8]	0.718	Kruskal

**Fig. 3:**
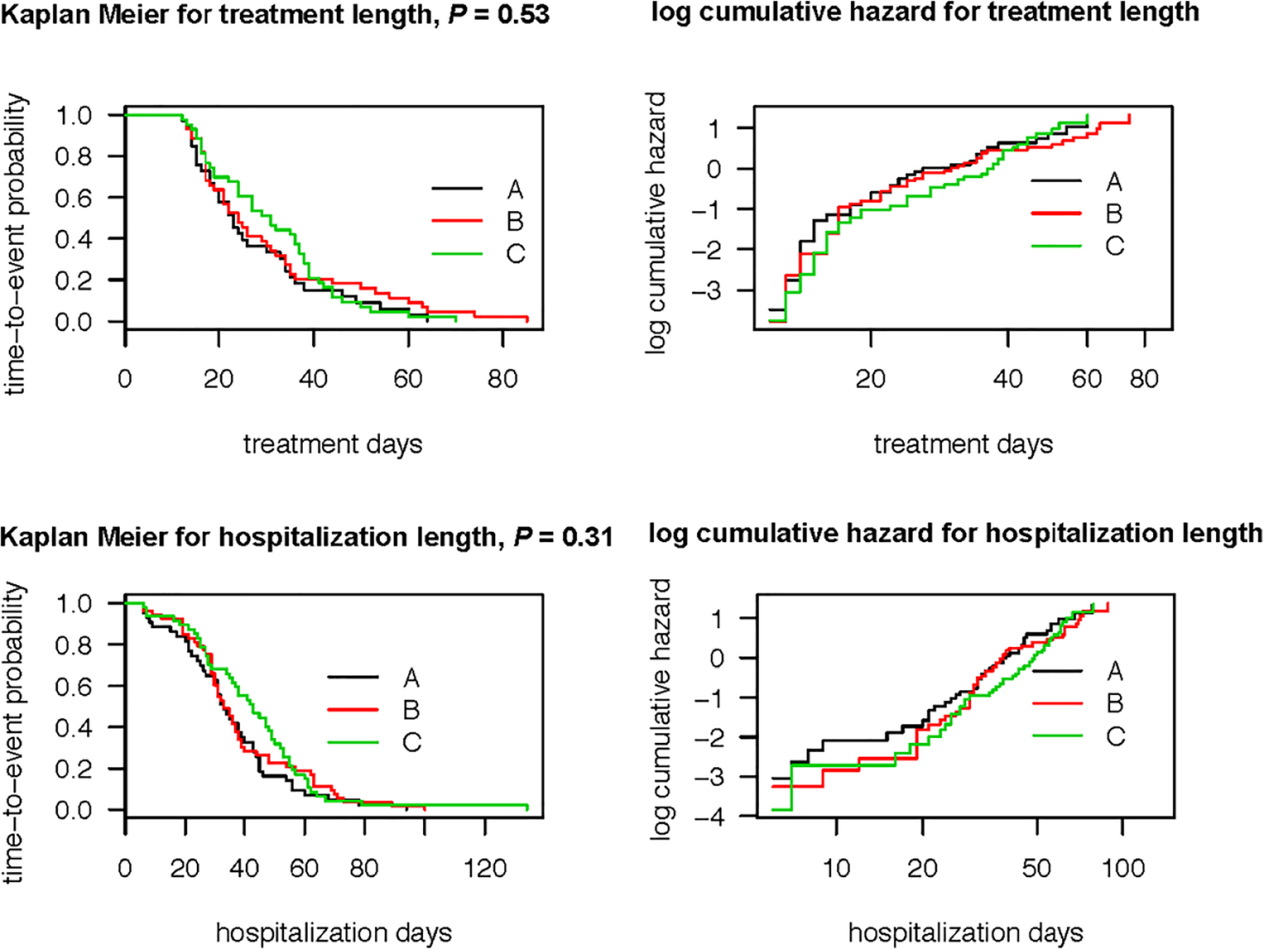
Kaplan-Meier and log cumulative hazard curves for treatment length and for hospitalization length. *p* values of a log-rank test are added. The time-to-event curves in the Kaplan-Meier plots can be interpreted as the complementary empirical distribution functions

There was no significant difference in length of medical treatment among groups. Median length of medical treatment in the morphine group was 22 days (95% CI from 18 to 33), in the chlorpromazine group 25 days (95% CI from 21 to 34), and in the phenobarbital group 32 (95% CI from 27 to 38 ) days (*p* = 0.12, Kaplan-Meier Curves in Fig. 3).

In the statistical analysis adjusted for sex, gestational age, small for gestational age (birth weight < 10th percentile), socioeconomic status, polydrug use of mother, and center, the chlorpromazine group had 17% longer treatment time (ETR 1.17, *p* = 0.15) and the phenobarbital group 16% longer treatment time than the morphine group (ETR 1.16, *p* = 0.19); neither difference was statistically significant.

Hospitalization time in the adjusted analysis was 5% longer in the chlorpromazine group than in the morphine group (ETR 1.05, *p* = 0.61) and 13% longer in the phenobarbital group than in the morphine group (ETR 1.13, *p* = 0.21). Neither difference reached statistical significance.

The need for a second medication differed considerably between the three groups. In the morphine group, only one out of 33 infants (3%) needed a second drug, but in the chlorpromazine group, the equivalent figure was 24 out of 44 (55%), and in the phenobarbital group, 13 infants out of 43 (30%) (Fisher’s exact test, *p* < 0.0001).

The majority of the mothers (71%) were multi-drug users. The meconium analyses detected amphetamine (*n* = 33 [27.5%]), barbiturate (*n* = 19 [15.8%]), benzodiazepine (*n* = 22 [18.3%]), cannabis (*n* = 27 [22.5%]), and cocaine (*n* = 28 [23.3%]) (Table 2).

**Table 2:** Results of meconium analyses in the tree groups

	Morphine (*n* = 33)		Phenobarbital (*n* = 44)		Chlorpromazine (*n* = 43)		Total	
	*n*	%	*n*	%	*n*	%	*n*	%
Amphetamine	12	36.4%	11	25.0%	10	23.3%	33	27.5%
Barbiturate	4	12.1%	8	18.2%	7	16.3%	19	15.8%
Benzodiazepine	7	21.2%	6	13.6%	9	20.9%	22	18.3%
Cannabis	4	12.1%	6	13.6%	17	39.5%	27	22.5%
Cocaine	7	21.2%	10	22.7%	11	25.6%	28	23.3%
Methadone	28	84.8%	43	97.7%	35	81.4%	106	88.3%
Opioid	17	51.5%	25	56.8%	32	74.4%	74	61.7%

Among the cofactors correlating with the primary outcome, only amphetamine in the meconium analysis was associated with a significantly shorter length of treatment (minus 7.6 days, *p* < 0.03, Table 3).

**Table 3:** Effect of drugs found in the meconium on treatment time in days

	Univariable		Multivariable	
Amphetamine	− 6.63	*p* = 0.04	− 7.652	*p* = 0.03
Barbiturate	− 2.03	*p* = 0.6	0.842	*p* = 0.84
Benzodiazepine	− 3.26	*p* = 0.37	− 3.145	*p* = 0.35
Cannabis	− 3.24	*p* = 0.34	− 1.476	*p* = 0.68
Cocaine	1.19	*p* = 0.72	4.442	*p* = 0.21

Linear model with univariable and multivariable analyses

The mean suffering score (sum of modified Finnegan scores > 9) was 238 (SD 180) for the morphine group, 277 (SD 152) for the chlorpromazine group, and 313 (SD 300) for the phenobarbital group. The analysis with adjusted and unadjusted models showed no treatment effect. In the 22 infants who did not need treatment, the mean suffering score was 14 (SD 28).

Intensity of care (nursing time) was 7 h per 24 h and did not differ significantly between the three groups in either the adjusted or the unadjusted model.

No seizures except tremor and sleep myoclonus, known to be associated with opioid withdrawal [9], were reported. Twelve infants (36%) in the morphine group, 13 infants (30%) in the chlorpromazine group, and 16 infants (36%) in the phenobarbital group showed at least one episode of hypothermia (rectal temperature below 36.0 °C or axillary temperature below 35.5°). No adverse events were observed that were not attributable to withdrawal.

## Discussion

The main finding of this trial is that none of the three drugs for controlling withdrawal symptoms in newborns to mothers who consumed opioids during pregnancy shortened treatment length compared with the other two. However, only one infant (3%) allocated to the treatment with morphine needed a second drug, whereas 55% of infants treated with chlorpromazine and 30% of infants treated with phenobarbital needed a second drug.

A systematic search in PubMed and Scopus found only a few controlled studies to compare with our results. Jackson et al. reported a shorter treatment length with morphine (*n* = 41, mean 8 days) than with phenobarbital (*n* = 34, mean 12 days, *p* = 0.04) [11], whereas Nayeri et al. found no significant difference in duration of treatment, duration of hospital stay, or requirement for adjunctive treatment between neonates with neonatal abstinence syndrome who received morphine sulfate and neonates who received a loading and tapering dose of phenobarbital [18]. A Cochrane review concluded that phenobarbital not only has a longer treatment length than morphine but is also associated with a higher incidence of seizures [20]. Therefore, it should no longer be used as a first-line drug [22].

The only trial comparing chlorpromazine with morphine used a historical control. Mazurier et al. found a mean length of 6 (range 3.5–9) days in infants treated with morphine, compared with 16 (10–21) days in infants treated with chlorpromazine (*p* < 0.001) [16]. Since this trial was published, safety concerns have been raised about chlorpromazine in infants younger than 6 months of age, and it therefore should no longer be used to treat neonatal abstinence syndrome.

In a recent meta-analysis including 18 trials, buprenorphine, a more potent analgesic than morphine, was suggested as the optimal treatment for neonatal abstinence syndrome. However, it concluded that limitations were considerable, and a large multisite trial of this treatment is required [6].

Another unresolved issue is dosing. Opioid pharmacokinetics vary considerably between infants [3]. Morphine has a short half-life and therefore has to be administered frequently, usually every 4 h. For blinding, the dose interval had to be the same for all three substances, even though this was not necessary for phenobarbital or chlorpromazine, as both have a longer half-life. Consequently, we started dosing with a relatively high dose of 0.25 mg per kilogram body weight, increasing stepwise up to 0.5 mg/kg (Fig. 1). The high initial dose was based on clinical experience that infants exposed in utero to an unknown quantity of opioids were better controlled with initial high dose and down-titration than a low initial dose and up-titration. However, this strategy did not allow us to observe respiratory insufficiency or other adverse effects. Down-titration could be started immediately after the first maintenance dose in two infants. Only one infant reached the predefined maximal dose of 0.5 mg/kg and therefore received a second drug.

We quantified suffering during withdrawal by calculating the area under the curve defined by the sum of modified Finnegan scores above 9 for each 8-h monitoring period. The time spent with a modified Finnegan score above 9 is a valid measure for suffering, since it has been shown that healthy newborns without neonatal abstinence syndrome may present symptoms contributing to an elevated modified Finnegan score, but the 95th percentile never exceeds 8 [23]. This suffering score was similar for all three groups.

We also estimated the need for staff resources by calculating the time that nurses spent with one infant. This was between 7 and 8 h per 24 h for all three groups, which is more than the mean nursing time (6 h per 24 h) in an intermediate care unit.

No adverse events other than those monitored by the Finnegan score were observed in our study, including seizures other than benign sleep myoclonus; this is known to be associated with both, morphine (12) and withdrawal itself. Episodes of hypothermia, known as a side effect of chlorpromazine, were documented in all three groups to the same extent and quickly resolved after warming the infant.

Among the potential confounders for length of treatment, only amphetamine in the meconium was positively correlated (Table 3). We did not expect this finding and could not find a similar observation in the literature. As amphetamine increases dopamine release and is sympathomimetic, we speculate that they may shorten neonatal abstinence syndrome. The exact mechanism needs to be elucidated. We would not recommend giving amphetamines to pregnant women consuming opioids in order to shorten neonatal abstinence syndrome, since the short-term and long-term adverse effects of prenatal amphetamine exposure are well documented in both, animal studies and human observational studies, including behavior problems at school age [7].

Strengths of this study are the consistent blinding of all health professionals, parents, and statisticians involved in the trial and the adherence to a strict treatment protocol, including tapering medication. It is a rare example of a well-designed and well-performed clinical trial in the vulnerable population of newborns [1, 15].

A weakness of the study is that infants were randomized before they reached a high modified Finnegan score for logistical reasons, so some did not need pharmacological treatment and were analyzed separately. The fact that neither opioid exposure nor exposure to other drugs during pregnancy could be quantified is also a potential problem, since the degree of drug exposure in utero may affect weaning symptoms.

Finally, this study was performed between the years 2001 and 2007, when drugs such as chlorpromazine and phenobarbital were used as first choice for neonatal abstinence syndrome whereas morphine was avoided to limit exposure after birth. Due to the expiry of funds and the promotion of the principal investigator to another position, data were not analyzed promptly. When reviewing the data in 2017, we concluded that publication of these data would still be of value, mainly because blinded, randomized data on neonatal abstinence syndrome treatment is sparse.

## Summary and conclusions

None of the tested drugs to treat neonatal abstinence syndrome resulted in a significantly shorter treatment length than the others. As morphine was able to control symptoms in almost all infants without adjuvant therapy, it may be preferred to the two other compounds but has to be tested against more potent opioids such as buprenorphine.

## Abbreviations

CI: Confidence interval
ETR: Event–time ratio
LR: Likelihood ratio
